# *MAP3K1* rs889312 polymorphism and cancer prognosis: A systematic review and meta‐analysis

**DOI:** 10.1002/cnr2.1773

**Published:** 2022-12-22

**Authors:** Md. Abdul Aziz, Mohammad Safiqul Islam

**Affiliations:** ^1^ Department of Pharmacy, Faculty of Pharmacy and Health Sciences State University of Bangladesh Dhaka Bangladesh; ^2^ Department of Pharmacy, Faculty of Science Noakhali Science and Technology University Noakhali Bangladesh; ^3^ Laboratory of Pharmacogenomics and Molecular Biology, Department of Pharmacy Noakhali Science and Technology University Noakhali Bangladesh

**Keywords:** cancer, *MAP3K1*, meta‐analysis, polymorphism, prognosis, survival

## Abstract

**Background:**

Accumulating studies have evaluated the association between *MAP3K1* polymorphisms and cancer prognosis. However, the results of these studies are conflicting. Given the potential impact of *MAP3K1* rs889312 SNP on the prognosis of various cancers, this meta‐analysis was performed to obtain solid and credible evidence.

**Methods and Materials:**

This study was performed according to the PRISMA 2020 statement. A comprehensive article search was conducted to find and select articles from multiple databases, including PubMed, Google Scholar, Web of Science, EMBASE and the Cochrane Library, published up to 15th September 2022. The data analysis was performed with Review Manager v5.2. Pooled HR with its 95% CI and *p*‐value was calculated where HR >1 suggests worse/poor survival and HR <1 suggests better survival of cancer patients.

**Results:**

A total of five articles comprising 24 439 patients were included for both qualitative and quantitative data synthesis. It was found that only the dominant genetic model (AC + CC vs. AA) showed a statistically significant poor overall survival for *MAP3K1* rs889312 polymorphism (HR = 1.25, 95% CI = 1.06–1.47, *p* = .01). In addition, publication bias analysis by the Egger's test and the Begg‐Mazumdar test reported no significant bias in the analysis of overall survival (*p* > .05).

**Conclusions:**

The present study concludes that *MAP3K1* gene rs889312 polymorphism plays a prognostic role in the survival of cancer patients. However, future research is recommended that will analyze more *MAP3K* SNPs along with rs889312, which may reveal more credible outcomes in terms of cancer prognosis.

## 1 INTRODUCTION

Despite groundbreaking advances in cancer research and treatment over the past decades, the rate of cancer incidence and mortality is continuously increasing, which has challenged global public health. It was found that the number of new cancer cases was more than 19 million in 2020, with a related death of around 10 million people.^1^, ^2^, ^3^ In the USA alone, it is supposed that the number of new cancer cases and deaths will be 1 918 030 and 609 360, respectively in 2022.^4^ Moreover, the 2040 projection by the researchers says that this trend will continue to rise and will reach around 30 million cancer incidences.^5^ The unbridled rate of cancer‐associated morbidity and mortality predisposes urgent actions to be taken regarding novel biomarkers to facilitate the evaluation of therapeutic outcomes for prolonged survival in cancer patients.^6^

**FIGURE 1:**
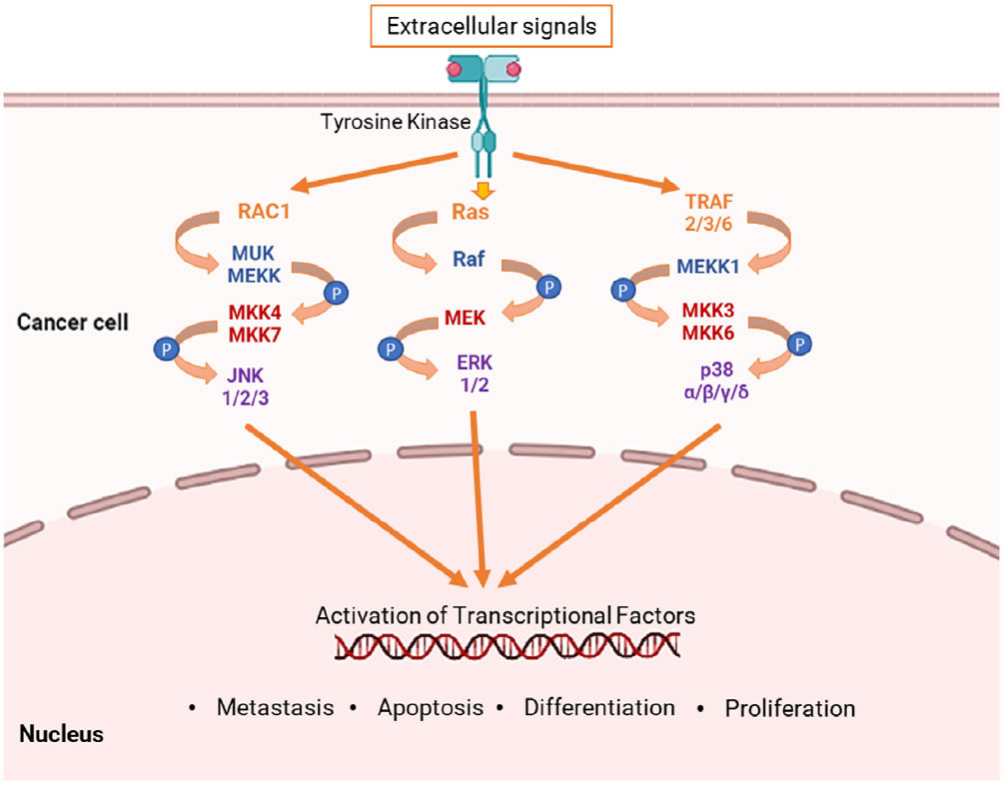
MAPK signaling pathways in cancer

Generally, mitogen‐activated protein kinases (MAPKs) are ubiquitously expressed and highly preserved in eukaryotes.^7^, ^8^ Numerous biological processes, including cell growth, proliferation, differentiation, migration, metabolism, and apoptosis, have been shown to be significantly influenced by the MAPK signaling pathways. Besides, this pathway is responsive to both intracellular signals (like cytokines, hormones, and growth factors) and extracellular/environmental signals. Therefore, abnormalities in the MAPK pathways alter the normal cellular processes that lead to the development and progression of multiple cancers (Figure 1).^7^, ^9^, ^10^ Different types of MAPK signaling pathways have been reported so far, including Big MAP kinase‐1, c‐Jun N‐terminal kinase (JNK), extracellular signal‐regulated kinase (ERK), and p38. The relationship between these pathways and chemotherapeutic agents has already been established.^11^, ^12^, ^13^ For instance, the MAPK/ERK signaling has been found to better the survival rate of cisplatin‐treated squamous carcinoma cells,^14^ and the P38α pathways have been reported to influence resistance to 5‐fluorouracil and cisplatin‐treated colorectal cancer patients.^15^

**FIGURE 2:**
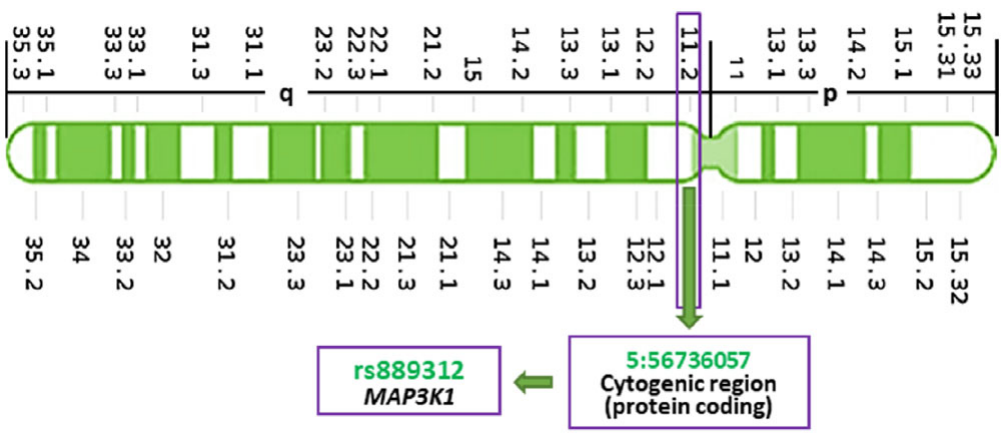
Chromosomal location of *MAP3K1* rs889312 polymorphism

Mitogen‐activated protein kinase kinase kinase 1 (MAP3K1) is a serine/threonine kinase that takes part in the MAPK transduction mechanism involving ERK, MEK, RAF, and RAS in response to a variety of metabolic and mitogenic components.^16^, ^17^ MAP3K1 has a pivotal impact on phosphorylation, and it activates MAPK2 following phosphorylation which then phosphorylates MAPK/ERK again to generate downstream signaling on tumor genes.^18^ GWAS and case–control association studies have shown that SNPs in the *MAP3K1* gene influence the development and progression of cancer in patients. The SNP rs889312, found on chromosome 5q11.2 (Figure 2) and mapped in the linkage disequilibrium (LD) block of *MAP3K1*, was first identified in 2007 by a GWAS.^19^ To date, a plethora of studies have reported the influence of *MAP3K1* rs889312 polymorphism on the prognosis of cancers, including distant disease‐free survival, disease‐free survival, or overall survival of breast cancer,^16^, ^20^, ^21^ colorectal cancer,^22^ gastric cancer.^23^ It was also reported that the rs889312 polymorphism is significantly linked with the risk of larger mammary tumors in Asian populations.^24^

Given the potential impact of *MAP3K1* rs889312 SNP on the prognosis of multiple cancers, we performed this meta‐analysis for the first time to obtain solid and credible evidence. We have focused on the studies that evaluated the correlation between survival outcomes of cancer patients and *MAP3K1* rs889312 polymorphism and tried to develop rs889312 as a prognostic marker.

## 2 METHODS AND MATERIALS

### 2.1 Data sources and search strategy

The present meta‐analysis was performed following the criteria defined in the latest Preferred Reporting Items for Systematic reviews and Meta‐Analyses (PRISMA) 2020 statement.^25^ A comprehensive article search was conducted to identify and select articles that reported the influence of *MAP3K1* rs889312 polymorphism on cancer prognosis from multiple electronic databases, including PubMed, Google Scholar, EMBASE, Web of Science, and the Cochrane Library, published up to 15th September 2022. Only the articles published in English were searched based on the predefined search terms: “*MAP3K1*/Mitogen‐Activated Protein Kinase Kinase Kinase 1”, “rs889312”, “Single nucleotide polymorphism/SNP/polymorphisms/variants/”, “Association/correlation/influence/link”, “Cancer/carcinoma/malignancy/tumor”, “Prognosis/prognostic”, and “Survival/outcome”. The references of the selected articles and published reviews on this topic were also manually searched to find out any relevant missing articles.

### 2.2 Inclusion and exclusion criteria

Articles meeting the following eligibility criteria were selected for the meta‐analysis: (a) Evaluated the correlation between *MAP3K1* rs889312 polymorphism and cancer prognosis; (b) Calculated hazard ratios (HR) and corresponding 95% confidence intervals (CI) for defined genetic association models, and (c) Performed on human cancer patients. In contrast, the following criteria were considered for the studies to be excluded: (a) Lacked sufficient genotyping data for HR calculation; (b) Reviews, commentaries, or letter to editors; and (c) Performed on animal samples.

### 2.3 Study selection, data extraction, and quality assessment

For the selection of eligible studies, two authors were recruited (MAA and MSI). They extracted the necessary data independently using a predesigned data extraction MS Excel sheet and any form of disagreements between them were resolved by discussion. The authors extracted the following basic characteristics of the included articles: cancer types, genetic models, name of the first author, year of the study published, the origin of the study population, sample size, outcome, and the HR (with a corresponding 95% CIs). For the assessment of study quality, the authors used the Newcastle‐Ottawa Scale (NOS) described by Wells and colleagues.^26^ During the process, three core characteristics, including selection, comparability, and outcome of studies, were examined. The quality assessment was also completed independently by MAA and MSI. A quality score of more than 7 indicates high quality, between 4 and 6 indicates medium quality, and less than 4 indicates poor quality.

### 2.4 Data analysis

The data analysis was performed with Review Manager (RevMan) software v5.4 (The Cochrane Collaboration, Oxford, UK). The data of four genetic models, including codominant model 1 (AC vs. AA), codominant model 2 (CC vs. AA), dominant model (AC + CC vs. AA), and recessive model (CC vs. AA+AC), were collected and analyzed for the *MAP3K1* rs889312 polymorphism and cancer prognosis. The HR with corresponding 95% CI was also collected from included studies. Pooled HR with its 95% CI and *p*‐value was calculated where HR >1 suggests worse/poor survival and HR <1 suggests better survival of cancer patients. The statistical heterogeneity was analyzed by *Q*‐statistic and *I* ^2^ statistics. In terms of significant heterogeneity (*p* value <0.1 or *I* ^2^ > 50%), the random‐effects model was applied; otherwise, the fixed‐effects model was applied. Publication bias was analyzed by Egger's test^27^ and the Begg‐Mazumdar test.^28^ A *p* value of ≤.05 was considered statistically significant.

## 3 RESULTS

### 3.1 Characteristics of the included studies

**FIGURE 3:**
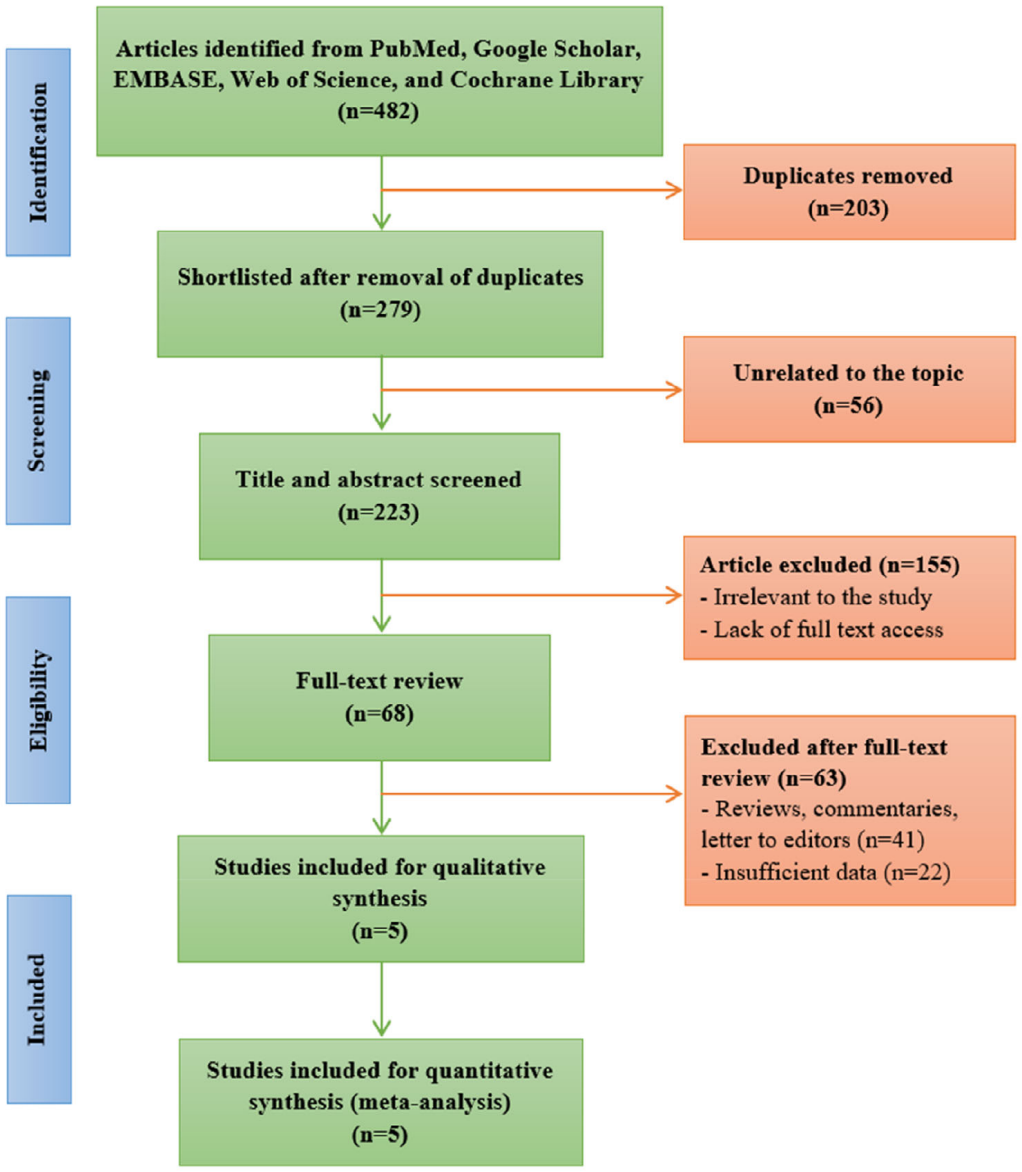
Flow diagram of the study selection

Figure 3 depicts the flowchart of the study selection method according to the PRISMA guidelines. Initially, a total of 482 articles from PubMed, Google Scholar, EMBASE, Web of Science, and the Cochrane Library were identified, and 279 articles were shortlisted after the removal of duplicates. After that, the title and abstract of 223 articles were screened and 155 were removed before the full‐text review of 68 articles. Due to not meeting the inclusion criteria, 63 articles were excluded, and finally, five articles^16^, ^29^, ^30^, ^31^, ^32^ comprising 24 439 patients were included for both qualitative and quantitative data synthesis. Of these articles, three studies were performed on the Chinese population^30^, ^31^, ^32^ from which the outcome and HR (with 95% CI) data of four genetic models, including codominant model 1, codominant model 2, dominant model, and recessive model were collected. One study was conducted on a mixed population^29^ and one study was performed on the Taiwanese population^16^ from which the data of the outcome and HR (with 95% CI) were collected for two genetic models (codominant model 1 and codominant model 2) and one genetic model (recessive model), respectively. Again, three articles were on breast cancer ^16^, ^29^, ^30^ and two were on gastric cancer.^31^, ^32^ The NOS quality score data revealed that all five included articles were of high quality (score ≥7). The basic characteristics of the included articles in this meta‐analysis are summarized in Table 1.

**TABLE 1:** Basic characteristics of the included articles in the meta‐analysis

Cancer types	Genetic models	Author et al.	Year	Study population	Sample size	Outcome	HR	95% CI lower	95% CI upper	NOS score
Gastric cancer	AC versus AA	Wei et al.	2014	Chinese	884	OS	1.30	1.01	1.68	8
	CC versus AA	Wei et al.	2014		884	OS	1.04	0.77	1.40	
	AC + CC versus AA	Wei et al.	2014		884	OS	1.18	0.94	1.49	
	CC versus AA+AC	Wei et al.	2014		884	OS	0.82	0.65	1.05	
Gastric cancer	AC versus AA	Yang et al.	2019	Chinese	371	OS	1.43	0.99	2.08	7
	CC versus AA	Yang et al.	2019		371	OS	1.01	0.65	1.56	
	AC + CC versus AA	Yang et al.	2019		371	OS	1.27	0.89	1.81	
	CC versus AA+AC	Yang et al.	2019		371	OS	0.795	0.56	1.13	
Breast cancer	AC versus CC	Fu et al.	2018	Chinese	343	OS	1.09	0.86	1.39	7
	AA versus CC	Fu et al.	2018		343	OS	0.78	0.51	1.11	
	AC + AA versus CC	Fu et al.	2018		343	OS	1.02	0.805	1.285	
	AA versus CC + AC	Fu et al.	2018		343	OS	0.74	0.54	1.01	
Breast cancer	AC versus AA	Fasching et al.	2012	Mixed	22 427	OS	0.99	0.93	1.06	7
	CC versus AA	Fasching et al.	2012		22 427	OS	0.97	0.86	1.09	
Breast cancer	CC versus CA + AA	Kuo et al.	2017	Taiwanese	414		2.10	1.10	3.80	8

Abbreviations: HR, hazard ratio; NOS, Newcastle‐Ottawa Scale.

### 3.2 Quantitative data synthesis of MAP3K1 rs889312 on cancer prognosis

**FIGURE 4:**
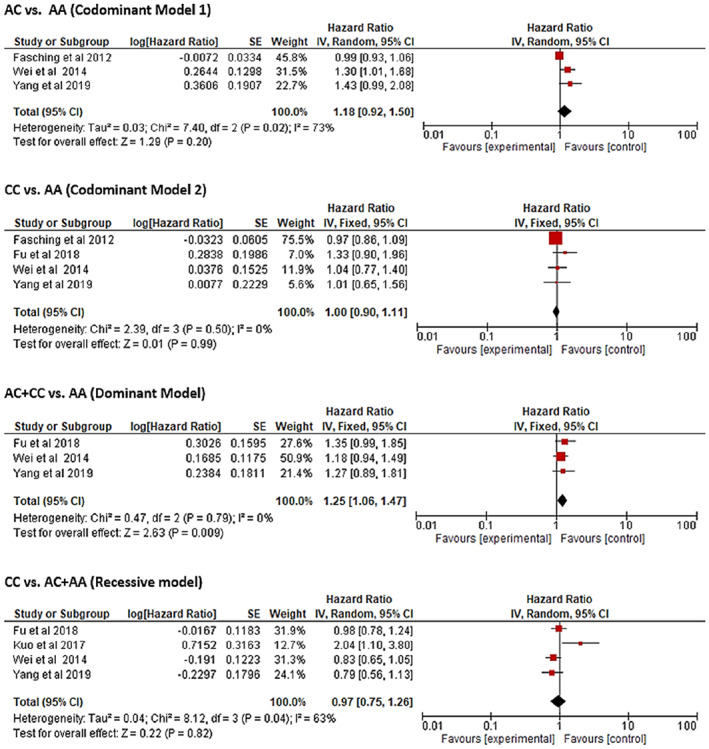
Forest plots for detecting the *MAP3K1* rs889312 polymorphism and cancer prognosis in different genetic models

Table 2 and Figure 4 explain the influence of *MAP3K1* rs889312 polymorphism on cancer prognosis. The pooled HRs and 95% CIs from the meta‐analysis for overall survival (OS) were calculated based on the four above‐mentioned genetic association models. Among the included articles, codominant model 1 was reported in three studies with 23 683 patients. The analysis revealed the codominant model 1 (AC vs. AA) of rs889312 polymorphism is associated with poor OS (HR = 1.18, 95% CI = 0.92–1.50, *p* = .20) but the association is not statistically significant. Codominant model 2 (CC vs. AA), on the other hand, was analyzed in 4 studies with 24 025 patients. However, this genetic model also showed a statistically non‐significant poor OS (HR = 1.00, 95% CI = 0.90–1.11, *p* = .99). Three relevant articles comprised of 1598 patients were analyzed in the dominant genetic model (AC + CC vs. AA) that showed a statistically significant poor OS for rs889312 (HR = 1.25, 95% CI = 1.06–1.47, *p* = .01). A total of four studies included the recessive (CC vs. AC + AA) model with 2012 patients. This genetic model showed a better OS, but the outcome was not significant (HR = 0.97, 95% CI = 0.75–1.26, *p* = .82).

**TABLE 2:** Pooled hazard ratios and 95% CIs from the meta‐analysis for overall survival

Genetic models	No. of studies	No. of subjects	HR (95% CI)	*p* value	Heterogeneity *I* ^2^ (%)	*p* value	Egger's test (*p* value)	Begg‐Mazumdar test (*p* value)
AC versus AA	3	23 682	1.18 (0.92–1.50)	.20	73	**.02**	.062	.602
CC versus AA	4	24 025	1.00 (0.90–1.11)	.99	0	.50	.269	.497
AC + CC versus AA	3	1598	1.25 (1.06–1.47)	**.01**	0	.79	.434	.602
CC versus AC + AA	4	2012	0.97 (0.75–1.26)	.82	63	**.04**	.318	.497

Note: Bold *p*‐values indicate statistically significant (*p*<.05).

### 3.3 Heterogeneity and publication bias

No statistically significant heterogeneity was observed for codominant model 2 (*I* ^2^ = 0%, *p* = .50) and the dominant model (*I* ^2^ = 0%, *p* = .79). In contrast, codominant model 1 and recessive model showed statistically significant heterogeneity (*I* ^2^ = 73%, *p* = .02 and *I* ^2^ = 63%, *p* = .04, respectively). Publication bias analysis by Egger's test and Begg‐Mazumdar's test reported no significant bias in the analysis of OS (*p* > .05).

## 4 DISCUSSION

This is, to the best of our knowledge, the first systematic meta‐analysis to evaluate the influence of *MAP3K1* rs889312 polymorphism on cancer prognosis. This study included five published articles involving 24 439 patients from different countries. The present study results indicated that *MAP3K1* rs889312 polymorphism might be associated with poor OS in cancer patients.

Established evidence suggests that MAPK cascades as the central signaling pathways that regulate a variety of key cellular processes such as apoptosis, differentiation, proliferation, and stress responses.^33^, ^34^ MAP3K1 or MEKK1 is a notable serine/threonine kinase characterized by its role in the signal transduction mechanism. It is established as a key player in the MAPK cell‐signaling cascade of phosphorylating enzymes that show response to a plethora of metabolic and mitogenic factors, for example, estrogen.^35^ The *MAP3K1* gene has a pivotal role in the MAPK‐dependent signaling mechanism that regulates the transcription of vital neoplastic genes, and the link between unregulated MAPK‐associated pathways with cancer has already been established. GWAS has discovered the SNP rs889312 genetic polymorphism closely positioned to the *MAP3K1* gene. The rs889312 was found to be located in an LD block of nearly 280 kb. It was demonstrated that this variant encodes a serine/threonine kinase and helps to assemble the part of the MAPK pathway characterized by cellular activities to mitogens.^19^, ^36^

To date, a wide variety of studies have explored the correlation between the *MAP3K1* rs889312 variant and the risk of cancer progression or prognosis. Due to the inconsistencies of previous studies, this meta‐analysis was performed. We have included five studies where three studies were from the Chinese,^30^, ^31^, ^32^ one study was from mixed,^29^ and one study was from Taiwanese population.^16^ We have collected the HR calculated for different genetic models from these studies and analyzed the effect of rs889312 on cancer prognosis. We have calculated pooled HR with 95% CI for OS, which demonstrated that only the dominant model showed a significantly poor OS for rs889312 (AC + CC vs. AA: HR = 1.25, *p* = .01). Although codominant model 1 (AC vs. AA) and codominant model 2 (CC vs. AA) showed poor OS (HR ≥1.00), these associations were not significant (*p* > .05). Although the recessive (CC vs. AC + AA) model depicted a better OS, the risk was not statistically significant (HR <1 and *p* > .05). Wei et al.^31^ and Yang et al.^32^ demonstrated that the *MAP3K1* rs889312 polymorphism might be considered a prognostic biomarker for gastric cancer development. Fu et al.^30^ also showed that rs889312 polymorphism substantially influences the prognosis of breast cancer.

*MAP3K1* gene rs889312 polymorphism has been extensively studied in breast cancer. A meta‐analysis reported that the C allele of rs889312 is a low‐penetrance susceptibility factor for breast cancer progression.^37^ Another study suggested that the SNP rs889312 was linked with a significantly increased risk of estrogen receptor‐negative breast cancer.^38^ On the other hand, Slattery et al.^39^ characterized that *MAP3K1* was not correlated with the susceptibility of breast tumors in American Hispanic and non‐Hispanic women. In the present study, three articles on breast cancer prognosis were included from Chinese,^30^ Taiwanese,^16^ and mixed population,^29^ which demonstrated a variable association between rs889312 polymorphism and OS of breast cancer. However, based on these inconsistencies, we have conducted the present study incorporating available studies that evaluated the association between cancer prognosis and rs889312 polymorphism.

In the present study, all the included articles were of excellent quality according to the NOS score. We have also performed heterogeneity and publication bias analyses for all genetic models applied. Although we observed heterogeneity for codominant model 1 and recessive model, no significant heterogeneity was reported for codominant model 2 and dominant model. The Egger's test and the Begg‐Mazumdar test confirmed the absence of any potential publication bias in the analysis (*p* > .05).

This study possesses some potential confounders that should be addressed. Firstly, there is a small number of studies incorporated in this meta‐analysis. Secondly, we have observed some sort of heterogeneity in the pooled HR analysis for OS. Thirdly, we did not perform sensitivity analysis due to the lower number of studies. Finally, more missing baseline characteristics should be included due to the lack of precise data in the included articles.

## 5 CONCLUSION

The present study concludes that *MAP3K1* gene rs889312 polymorphism plays a prognostic role in the survival of cancer patients. More precisely, the dominant model is significantly associated with poor overall survival for rs889312 in cancer. Future studies are recommended that will analyze more *MAP3K* SNPs along with rs889312, which may reveal more credible outcomes in terms of cancer prognosis.

## AUTHOR CONTRIBUTIONS

**Md. Abdul Aziz:** Data curation (equal); methodology (equal); resources (equal); writing – original draft (equal); writing – review and editing (equal). **Mohammad Safiqul Islam:** Conceptualization (lead); formal analysis (lead); software (lead); writing – original draft (equal); writing – review and editing (equal).

## CONFLICT OF INTEREST

The authors have stated explicitly that there are no conflicts of interest in connection with this article.

## ETHICS STATEMENT

Not required as it is a meta‐analysis that anlayzed the published data.

## Data Availability

On request, the corresponding author will make available all data used in this work.
